# NeuroNet19: an explainable deep neural network model for the classification of brain tumors using magnetic resonance imaging data

**DOI:** 10.1038/s41598-024-51867-1

**Published:** 2024-01-17

**Authors:** Rezuana Haque, Md. Mehedi Hassan, Anupam Kumar Bairagi, Sheikh Mohammed Shariful Islam

**Affiliations:** 1 Computer Science and Engineering, BGC Trust University Bangladesh, Chittagong, Bangladesh; 2 Computer Science and Engineering Discipline, Khulna University, Khulna, 9208 Bangladesh; 3 Institute for Physical Activity and Nutrition, Deakin University, Melbourne, VIC 3125 Australia

**Keywords:** Computational biology and bioinformatics, Neuroscience, Neurology

## Abstract

Brain tumors (BTs) are one of the deadliest diseases that can significantly shorten a person’s life. In recent years, deep learning has become increasingly popular for detecting and classifying BTs. In this paper, we propose a deep neural network architecture called NeuroNet19. It utilizes VGG19 as its backbone and incorporates a novel module named the Inverted Pyramid Pooling Module (iPPM). The iPPM captures multi-scale feature maps, ensuring the extraction of both local and global image contexts. This enhances the feature maps produced by the backbone, regardless of the spatial positioning or size of the tumors. To ensure the model’s transparency and accountability, we employ Explainable AI. Specifically, we use Local Interpretable Model-Agnostic Explanations (LIME), which highlights the features or areas focused on while predicting individual images. NeuroNet19 is trained on four classes of BTs: glioma, meningioma, no tumor, and pituitary tumors. It is tested on a public dataset containing 7023 images. Our research demonstrates that NeuroNet19 achieves the highest accuracy at 99.3%, with precision, recall, and F1 scores at 99.2% and a Cohen Kappa coefficient (CKC) of 99%.

## Introduction

The brain is the heaviest and most sensitive organ in the human body. It is responsible for almost every function of our body. Functions such as movement of the body parts, taste, hearing and seeing, sensation, smell, etc. are all controlled by the brain. A BT is an abnormal growth of brain cells within the brain. Tumors in the brain can hamper the functions of brain tissues and put unwanted pressure on nearby regions. BT can affect the regular flow of cerebral fluid or even cause bleeding^1^. As a result, the pressure inside the skull may increase, and there may be fluid accumulation. Tumors are various in size and can occur in any location of the brain. There are 120 types of BTs^2^. The complex structure of the brain makes detection and treatment difficult. The symptoms of the BT depend on its location, size, and origin^3^. The most common symptoms include severe headaches, unplanned seizures, and abnormalities in hearing, smell, and vision. Other symptoms are a shift in personality, trouble sleeping, struggle with memory retention, feeling perpetually tired, or suffering from nausea and vomiting^1^. Symptoms can vary depending on the location of the tumor, including vision problems, communication difficulties, hearing difficulties, swallowing difficulties, motor skill issues, balance issues, and facial paralysis or weakness^4^. However, it is always recommended to consult with a medical professional for a robust diagnosis.

BTs are primarily divided into two types based on their origin in the brain: primary BTs and secondary BTs. Primary BTs occur in the brain, which means they originate from the brain^5^. Secondary BTs originate from the brain, and they subsequently spread to the other organs of the body^5^. BTs can be further classified based on their malignancy^6,7^. The term “malignancy” refers to the presence of cancerous cells. Benign tumors are non-cancerous tumors. These tumors are problematic, and they put pressure on the neighboring cells. Benign tumors are less harmful, and they are less likely to spread. On the other hand, malignant tumors are cancerous in nature, and they can spread to other regions of the brain or even to other parts of the body^8,9^. These tumor cells grow and multiply. This behavior poses a serious threat to human health if the tumor doesn’t stop growing or get removed. Surgery, radiation therapy, and chemotherapy are frequently used for the treatment of malignant BTs.

Brain and central nervous system (CNS) cancer is one of the leading causes of death^10^. According to previous studies, CNS cancer will affect close to 18,990 people (11,020 men and 7970 women), according to cancernet2023brainstats. In 2020, 251,329 people died globally due to primary cancerous brain and CNS tumors^10^. According to the publication by the American Cancer Society, about 3490 new cases of brain and CNS cancer are estimated in 2023; among these new cases, 1900 are males and 1590 are females^11^. Early detection and accurate classification are crucial, but also very challenging. The world of neurology is continuously striving to make improvements in this regard.

Neuronal magnetic resonance imaging (MRI) provides a clear and detailed view of the brain image. An MRI scan plays an important role in recognizing BTs^12^. In this process, a special dye is injected to enhance the MRI image quality, which helps to distinguish between tumors and healthy tissues. Functional MRI (fMRI) has the ability to describe which part of the brain is responsible for a specific task. This helps to plan treatment for various brain diseases, like BTs. Magnetic resonance spectroscopy also measures the chemical levels in tumors. This helps to classify the specific BT type. However, manually examining brain MRI scans by considering all these points is a challenging and time-consuming task for healthcare experts like clinicians, doctors, and radiologists. The use of a computer-aided diagnosis model for BT classification will make the job simpler for doctors and save them a lot of time.

In the area of BT detection, many studies use image processing and transfer learning methods to improve the model’s accuracy. The main challenges in this field are the variety of tumor types, the spatial positioning of the tumors, and the imbalanced or limited dataset. Tumors can be large or small, and they can appear in any portion of the brain. This makes it hard for the model to classify tumor types. Many datasets in this field are either limited or imbalanced, resulting in biased learning. These challenges are evident in recent literature. Some studies^13,14^ reported low accuracy in their models. The datasets used in some research^15,16^ were either limited in volume or had significant class imbalances. This could affect the performance of the model. Our model, NeuroNet19 accurately classified four tumor types, but a study^17^ had difficulty achieving good accuracy even with a simpler three-group model. There isn’t any information in these papers about how to deal with challenges like tumor sizes and location variability. Furthermore, it’s not clear which specific features their models are using to make their predictions. However, one study^18^ employed the layer-wise relevance propagation (LRP) method and utilized heatmaps for model interpretability. Despite using this method, their accuracy is low compared to our model. Also, they skipped pre-processing the data and instead focused on a smaller class-based training method. Addressing these challenges may lead to more robust and accurate MRI brain tumor detection systems. Our research seeks to fill these gaps. The goal is to improve BT detection in MRI scans by developing a model that considers tumor sizes, locations, and imbalanced datasets while maintaining model transparency.

In our research, we’ve introduced a model called NeuroNet19. This model is a modified version of the VGG19 architecture. We have expanded the VGG19 architecture by adding a module named Inverted Pyramid Pooling Module (iPPM). The iPPM contributes to the model’s performance by providing a unique set of operations that improve the model’s ability to extract important features from the images. These changes aim to improve the model’s classification of BT images. Our research presents the first attempt to apply the iPPM method to medical imaging, and it shows great promise for future improvements in this field. This study outperforms past studies by achieving an impressive accuracy rate of 99.2%.

The contributions of our work can be summarized as follows:

1. *Adaptation of VGG19 for Brain Tumor Classification with iPPM:* In our research, we introduce a modified version of the VGG19 architecture, combined with iPPM. VGG19 is renowned for its image recognition capabilities. By merging the VGG19 architecture with iPPM, we aim to provide a more comprehensive feature representation, thereby countering these challenges.
2. *Addressing Brain Tumor Classification Challenges with iPPM:* BT classification has some challenges, such as complicated tumor patterns, an imbalanced dataset, spacial positioning of the tumors, and multiple class variances. The iPPM, with its unique pooling approach, can capture both macro and micro-features within the images. To overcome the challenge of the imbalanced dataset, we used a dataset that is comparatively balanced and large in number.
3. *Pioneering Application of NeuroNet19:* To our understanding, this research represents the first-ever attempt to combine VGG19 with the Inverted Pyramid Pooling (iPPM) technique. This method signifies a unique advancement in the field of medical image analysis. Our research presents a new perspective on BT classification, leveraging the combined strength of VGG19 and iPPM.
4. *Enhanced Feature Extraction for Medical Imaging:* The VGG19 architecture is known for its deep layers and exceptional feature extraction capabilities. When VGG19 is combined with the multi-scale feature capture of iPPM, it offers a robust approach for BT image classification. This strategy is anticipated to capture detailed insights from MRI brain scans. This method is crucial for accurate diagnosis and medical interventions.

## Literature review

BT detection is of the utmost importance in medical imaging and healthcare. Researchers are motivated by this field due to the significance of early detection for effective treatment and improved patient outcomes. Deep learning techniques’ potential and the complexity of BTs drive researchers to create cutting-edge detection techniques.

A study^19^ presents a weighted average ensemble deep learning model for classifying BTs in MRI images. The researchers used a dataset from the Cancer Genome Atlas (TCGA) that included 3929 MRI images of patients with lower-grade glioma. Their ensemble model achieves superior performance with an accuracy of 98%, precision of 98.25%, and F1-score of 98.5% by combining feature spaces from three different models, and they optimized weights by grid search. It should be noted that the findings of the study may not apply to other types of cancer-attacking MRI images.

Using the Learning by Self-Explanation (LeaSE) architecture search method, a study^20^ created an automated technique for classifying BTs from MRI images. The LeaSE technique was used to classify 3264 MRI images into four categories: glioma, meningioma, pituitary tumor, and healthy. Their method outperformed the human-designed ResNet101, achieving 90.6% accuracy with 3.75 million parameters and 84.5% accuracy with 42.56 million parameters.

Another study^21^ describes a method for detecting BTs in magnetic resonance images (MRI) using a fine-tuned EfficientNet model. A deep convolutional neural network (CNN) based on the EfficientNet-B0 architecture is used in the proposed method. The dataset contains MRI images that are critical in identifying areas of tumor. Several image enhancement techniques (the use of filters) are used to improve image quality. The achieved validation accuracy of 98.87% showcases the high performance of their proposed model.

An article^22^ proposed a CNN model for BT classification that was integrated with a graph neural network (GNN). Their method addressed the challenges caused by non-Euclidean distances in image data and pixel similarity based on proximity. They used a public dataset from Kaggle. They trained a 26-layer CNN combined with graph convolution operations in their graph-based convolutional neural network (GCNN). This process modifies node features by aggregating information from neighboring nodes, improving tumor region representation. Net-2 achieved the highest accuracy of 95.01% among the five developed networks.

In a study^23^, the researchers developed a method for classifying multilevel BTs from MRI images using the Sine Cosine Archimedes Optimization Algorithm (SCAOA). The Gaussian filter was used to preprocess the MRI images obtained from BRATS 2020 and the Figshare Dataset. SegNet, a fusion of the Archimedes Optimization Algorithm (AOA) and the Sine Cosine Algorithm (SCA), was used to segment tumors. The following features were extracted: post-segmentation, statistical, Haralick, and texture. ShCNN, trained with the proposed SCAOA, was used to detect BTs. If the image was of a tumor, it was further classified into types such as pituitary tumors, gliomas, and meningiomas using SCAOA-tuned DenseNet. The final model achieved an impressive 93.0% accuracy, 92.3% sensitivity, and 92% specificity.

A research study^24^ developed ARM-Net (an attention-based residual multi-scale CNN) for BT classification from MRI images. To address the challenges posed by high inter-class similarities, the model employed a lightweight global attention module (LGAM) and focused on critical features. ARM-Net achieved 96.64% and 97.11% accuracy, respectively, on the MBTD and BraTS 2020 datasets.

Another study^25^ evaluated the use of two CNN models for MRI-based BT classification. They achieved up to 96% accuracy through hyperparameter optimization and data augmentation. To better understand how these models made decisions, they used explainable artificial intelligence (XAI) techniques.

In a study^16^, the authors proposed a deep learning approach for BT classification and segmentation based on a multi-scale convolutional neural network. They used 3064 slices of T1-weighted contrast-enhanced MRI images from 233 patients with meningiomas, gliomas, and pituitary tumors to make sure the model was correct. With an average Dice index of 0.828, a sensitivity of 0.940, and a pttas value of 0.967, their approach outperforms the competition in segmentation. However, it is important to note that the method may still produce false positives.

A study^26^ creates an artificial intelligence model for detecting BTs. They used ResNet50 as their model. On a dataset of 3064 MRI images, the model achieves 95.32% accuracy. However, the study has limitations, such as the lack of comparative testing and the small dataset.

A different article^15^ talks about a way to find BTs using a modified ResNet50 model and a histogram of gradient (HOG)-based feature extraction framework. The dataset they used consists of 253 MRI images obtained from Kaggle. The authors used a feature optimization approach to extract more intuitive features from the complex feature vector, yielding a hybrid model with improved computational efficiency. The proposed method used the HOG and modified ResNet50 models and achieved a detection accuracy of 88%. The study acknowledged limitations in detecting tumor region substructures and accurately classifying healthy and unhealthy images.

A research study^27^ proposed an approach for BT segmentation using MRI multi-modalities. Utilizing the BRATS 2018 dataset, the researchers introduced a cascade convolutional neural network combined with a distance-wise attention mechanism. This method achieved impressive dice scores for the whole tumor (0.9203), enhancing tumor (0.9113), and tumor core (0.8726), highlighting its potential for efficient BT segmentation.

The study^28^ presents the Transformer-Enhanced Convolutional Neural Network (TECNN), a hybrid deep learning-based model for effective BT classification using MRI images. The proposed TECNN model combines the strengths of CNNs and transformers for local feature extraction and self-attention mechanisms for capturing long-range dependencies. They utilized two publicly available datasets, BraTS 2018 and Figshare. The model achieves an average accuracy of 96.75% and 99.10%, respectively.

## Methodology

**Figure 1:**
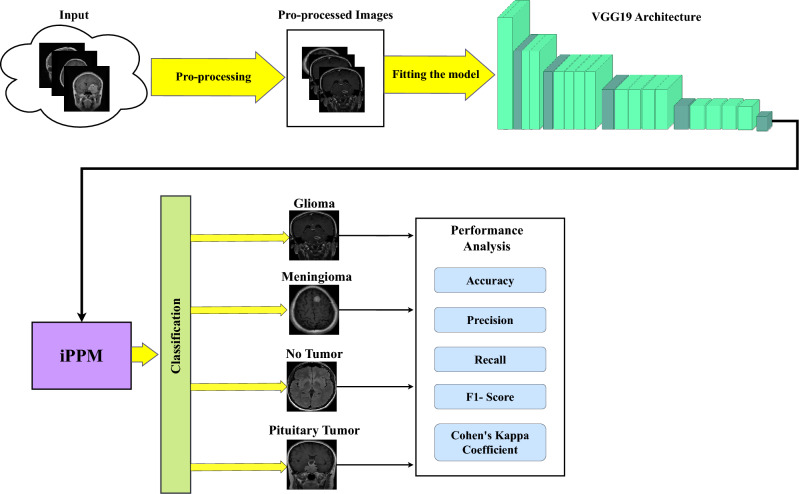
Data flow diagram of proposed model.

For better understanding, the whole process is illustrated in a data flow diagram in Fig. 1. Initially, we processed the raw images for more effective feature extraction. We employ multiple operations on the images, such as Gaussian blur, Otsu’s thresholding, contour finding, image cropping, and power-law transformation. These image enhancement techniques are applied to prepare the images for effective feature extraction. Following pre-processing, we have split the processed images into three subsets: training, validation, and testing. The training set is used to train the proposed model, NeuroNet19, the validation set for hyperparameter tuning, and the testing set to evaluate the model. Using the validation set helps to prevent overfitting and underfitting. The testing set, unknown to the model, allows us to assess how NeuroNet19 performs with new data.

The training set is increased with data augmentation. Data augmentation not only expands the dataset size but also enhances the diversity of the data. Data augmentation for NeuroNet19 includes rotating the images, horizontally flipping them, applying shear transformations, zooming in and out, as well as shifting their width and height. It should be noted that these augmentation techniques are only applied to the training set. The validation and test sets are left in their original, unaltered form to get a reliable and unbiased model. After pre-processing, the images are forwarded through a VGG19 neural network architecture. VGG19 is known for extracting complex features from images. This deep neural network architecture can capture hierarchical patterns in images and produce intermediate feature maps. However, these feature maps are not the endpoint; they are further refined and processed through iPPM. This iPPM module helps the model understand the features at different levels, making it better at classifying the images accurately.

Finally, NeuroNet19 is evaluated using multiple evaluation metrics. These metrics include the confusion matrix, classification report, accuracy score, precision, recall, F1-score, and lastly, CKC. Accuracy presents the ratio of the correct predictions out of all the predictions made by NeuroNet19. Precision shows how many predictions the model predicted as positive, and the recall states how many positive predictions our model NeuroNet19 correctly predicted. The F1 score takes precision and recall into account and presents the harmonic mean of these two. Lastly, CKC is used to measure the level of agreement between predicted and actual classifications.

### NeuroNet19 architecture (proposed model)

**Figure 2:**
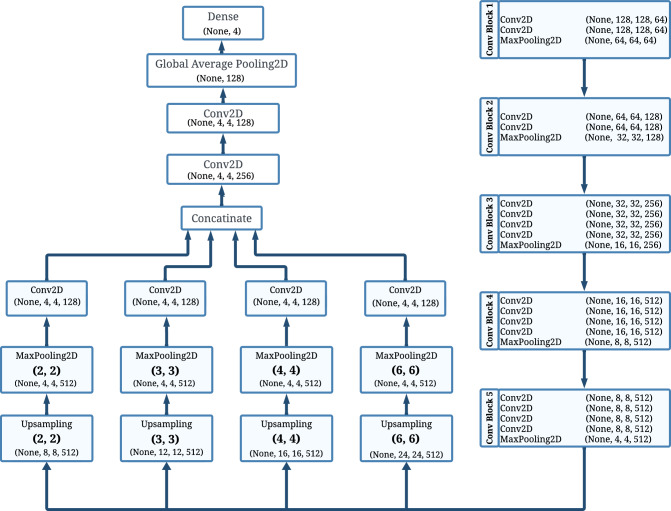
Model architecture of NeuroNet19.

The proposed NeuroNet19’s architecture is a combination of VGG19 and iPPM. In the NeuroNet19 model, VGG19 serves as the structural core or “backbone” of the system. The hierarchical structure of VGG19 makes the model capture both simple and complicated patterns within the images. This model works as the foundation for extracting essential features from image data; the iPPM then refines the feature maps in a better way so that the model classifies the instances accurately. Figure 2 shows the architecture of NeuroNet19.

#### The backbone of NeuroNet19

VGG19 is considered to be the central support structure of the NeuroNet19 for extracting the features. The backbone VGG19 was developed by the Visual Geometry Group (VGG) at the University of Oxford. It is popular for its efficiency and simple architecture. VGG19 is capable of capturing complex hierarchical features in images. It serves as the initial filter for NeuroNet19. VGG19 takes pre-processed image data and extracts the most important features from those images. For this reason, VGG19 is used as the backbone of NeuroNet19. It consists of 19 layers. There are five max-pooling layers, three completely linked layers, sixteen convolution layers, and one softmax function layer. It follows a specified order of the convolution, activation, and max-pooling layers. The convolution layer applies a set of filters or kernels across the input image or feature map from the previous convolution layer. The convolution operation is performed by sliding a filter F over the input image M. At each position (u, v), the following computation occurs:

1 $$\begin{aligned} C_{u,v} = \sum (F \odot M_{\text {sub}}) \end{aligned}$$

Here, $C_{u,v}$ represents the value at position $(u, v)$ in the output matrix $C$ and $M_{\text {sub}}$ refers to the area of the input matrix M that the filter covers at the current position. $F \odot M_{\text {sub}}$ denotes the element-wise product of the filter $F$ and the sub-matrix $M_{\text {sub}}$. The convolution operation involves the sum of element-wise products between the image and the kernel. Each convolution layer applies multiple filters, and each filter is designed to extract specific features or characteristics from the input data. As a result, the output of a convolution layer is a collection of feature maps made by various filters. The process of generating a single feature map can be expressed as follows:

2 $$\begin{aligned} O(L)_f = B(L)_f + \sum _{k=1}^{N(L-1)} F(L)_{fk} *O(L-1)_k \end{aligned}$$

In this process, $O(L)_f$ is the output of the $f{th}$ feature map in Layer $L$. Each filter $F(L)_{fk}$ of the current layer L is applied to all the feature maps of the previous layer L-1. In the Eq. (2), $O(L-1)_k$ is the output of the $k{th}$ feature map from the previous layer (Layer $L-1$). Here, $f$ refers to a specific filter in layer $L$, and $k$ indicates a particular feature map from the previous layer $L-1$ that this filter is applied to. The sign $*$ denotes the convolution operation between the filter $F(L)_{fk}$ from the current layer $L$ and the output $O(L-1)_k$ of the $k{th}$ feature map from the preceding layer $L-1$. Lastly, $B(L)_f$ represents the bias term associated with the $f{th}$ feature map in Layer $L$. After that, in Layer L, a new feature map is created by adding up all the result values. This procedure combines the data from all of the feature maps in the previous layer that the filter had captured. The output feature maps highlight various aspects or features detected in the input. The next critical step is the application of a nonlinear activation function to each feature map. This step introduces non-linearity and increases the network’s ability to learn and model complex patterns. The backbone (VGG19) uses ReLU (Rectified Linear Unit) as the activation function. The mathematical definition of the ReLU activation function is:

3 $$\begin{aligned} f(z) = \max (0, z) = {\left\{ \begin{array}{ll} 0 &{} \text {if } z < 0 \\ z &{} \text {if } z \ge 0 \end{array}\right. } \end{aligned}$$

For each element (z) in a feature map, ReLU sets all negative values to zero and keeps positive values as they are. This is an element-wise operation applied across the entire feature map. VGG19 consists of max-pooling layers that are used to reduce spatial dimension. The method divides feature maps into non-overlapping sections of size p$\times$q and selects the highest value from each. The max-pooling operation can be mathematically expressed as:

4 $$\begin{aligned} f_{\text {max}}(B) = max_{p \times q}(B_{p \times q}) \end{aligned}$$

The max pooling operation is applied to each p$\times$q region inside B, and $B_{p \times q}$ denotes each region within the input matrix B. The function $f_{\text {max}}(B)$ returns the greatest value discovered in each of these regions, resulting in a reduced spatial representation of the input matrix B. The depth of the VGG19 allows it to capture a wide range of complicated features. After extracting the features, the feature maps are sent to the iPPM to obtain the refined version of the features.

#### Inverted pyramid pooling module (iPPM)

When the VGG19 extracts the necessary feature maps from the images, iPPM steps in to refine these features. iPPM enhances feature extraction by employing various pool sizes as upsampling factors. The pool sizes include 2x2, 3x3, 4x4, and 6x6. The main concept of iPPM is to extract multi-scale feature maps to get a more comprehensive depiction of an input image. The process begins with the upsampling of feature maps. This step restores the spatial details that may have been lost during previous downsampling stages in the network. In NeuroNet19, we used the nearest neighbor upsampling method. The equation can be expressed as:

5 $$\begin{aligned} U(i, j) = I\left( \left\lfloor \frac{i}{s} \right\rfloor , \left\lfloor \frac{j}{s} \right\rfloor \right) \end{aligned}$$

In the Eq. (5), $I$ is the input tensor, and $U(i, j)$ represents the pixel value at position $(i, j)$ in the upsampled output. $s$ is the upsampling factor; for NeuroNet19, the upsampling factor is the pool sizes. It means we used different sizes of the upsampling factor. After upsampling, max pooling is applied to the upsampled tensor with the same spatial dimensions as the upsampling factor. Here, we used the nearest neighbor method for upsampling. This method increases the size of feature maps by repeating pixel values. This does not contribute additional information but rather changes the scale at which features are represented. Applying max pooling to these enlarged maps does reduce them back to their original size. However, because the features were enlarged first, the pooling operation now has a different contextual basis. The pooled tensor is then sent to the 1x1 Conv2D layer, which compresses the feature information and reduces the depth of the pooled tensor. For each pyramid level $i$:

6 $$\begin{aligned} F_i = \text {Conv}(\text {MaxPool}(\text {UpSample}(I, s_i)), k_i) \end{aligned}$$

Equation (6) represents the entire iPPM operation for a feature map. In the iPPM, each feature map $F_i$ at a specific pyramid level $i$ is generated through a series of operations. The process begins with upsampling the input tensor $I$ by a factor $s_i$, enlarging the tensor to capture more spatial detail. This upsampled tensor then undergoes max pooling, denoted as $\text {MaxPool}$, using the same scale factor, which emphasizes the most significant features within the enlarged regions. Finally, a convolution operation, represented as $\text {Conv}$, with a kernel size $k_i$ is applied to this pooled tensor. This sequence of upsampling, pooling, and convolution at different scales enables the iPPM to extract a rich, multi-scale representation of the input image. Finally, the outputs from the various pyramid levels $F_i$ are concatenated together. This step combines the features extracted at various scales. The concatenation of feature maps from various levels of the iPPM can be represented as follows:

7 $$\begin{aligned} C = \text {Concat}(F_1, F_2, F_3, \ldots , F_i) \end{aligned}$$

Here, $C$ is the concatenated output, and $F_1, F_2, F_3, \ldots , F_n$ are the feature maps from different levels of the iPPM that are being concatenated. The combination of several upsampling sizes followed by appropriate pooling enables the iPPM in NeuroNet19 to capture a wide range of characteristics. Each pooling size is designed to target sets of features found in the image. By applying pooling operations at different sizes, the iPPM module extracts information from both large and small regions within the image. The combination of these scales creates a comprehensive representation of the data, improving the model’s ability to extract and identify patterns, textures, and structures more effectively.

The feature representation is improved even more by the extra processing layers after the iPPM. Two Conv2D layers are applied, each with a 3x3 kernel size and 256 and 128 filters, respectively. After the convolution layers, a global average pooling 2D layer is applied. This layer calculates the mean value of each feature map in terms of the two spatial dimensions. The final dense layer outputs a probability distribution over classes using a softmax activation function and has the same number of units as classes. The equation for the softmax function is as follows:

8 $$\begin{aligned} \text {Softmax}(z_i) = \frac{e^{z_i}}{\sum _{j=1}^{K} e^{z_j}} \end{aligned}$$

The softmax function transforms network outputs into $K$-class probability distributions. Here, $z_i$ is the input for each class, and $e$ is Euler’s number. For each class, $e^{z_j}$ computes the exponential of the output value $z_j$ corresponding to that class. This summation makes sure that the softmax output for each class i (shown as $\text {Softmax}(z_i)$) is standardized compared to the outputs for all the other classes. This creates a probability distribution that is spread out across the classes.

In summary, the backbone provides a “knowledge base” to learn from. It extracts the most essential local and global image features as a backbone. Here, the iPPM serves as the magnifying glass. iPPM looks for the features that were missed by the backbone. It captures macro and micro-patterns by using a multi-scale approach. The pseudo-code for the complete NeuroNet19 architecture, including the iPPM module, is as follows.

***Initialize libraries and parameters*** 
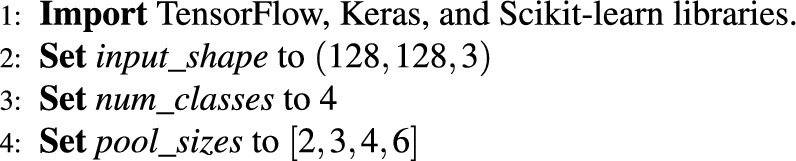


***Pyramid pooling module function*** 
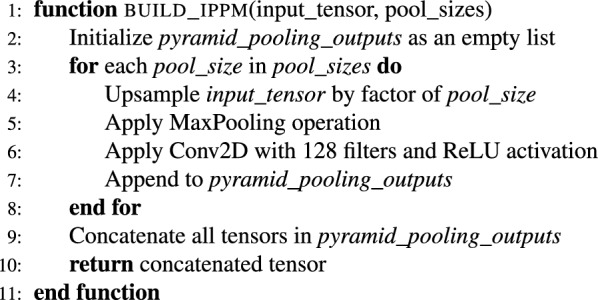


***Backbone function*** 
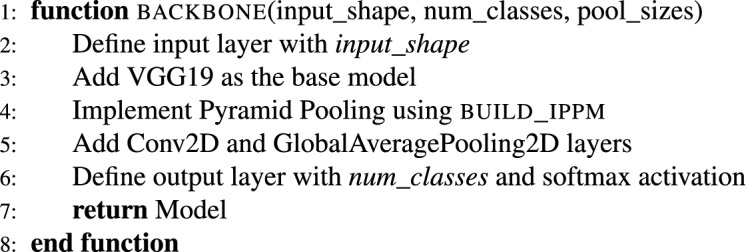


***Model initialization and compilation*** 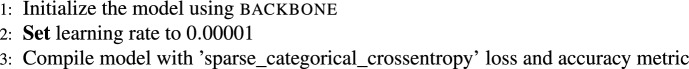


***Data augmentation*** 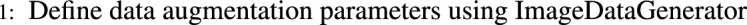


***Training with KFold*** 
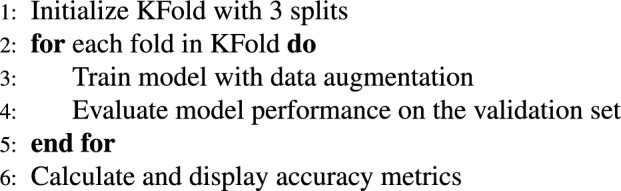


***Results and plotting*** 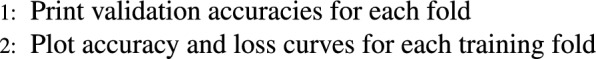


### Dataset

**Figure 3:**
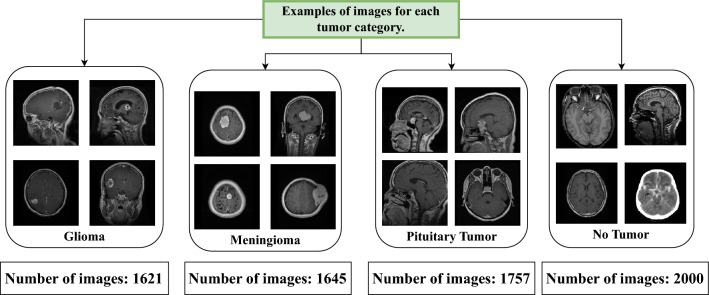
Sample images from each class.

In this research, the “Brain Tumor MRI Dataset”^29^ is employed. This dataset is publicly accessible and combines data from three distinct sources: Figshare, the SARTAJ dataset, and Br35H. This dataset contains 7,023 human brain MRI images and categorizes the images into four distinct classes: glioma, meningioma, no tumor, and pituitary. Figure 3 showcases representative images from each of the four classes: glioma, meningioma, pituitary tumor, and no tumor. These pictures illustrate the specific features that define each category.

1. *Glioma:* The glioma images typically display irregularly shaped masses with heterogeneous intensity, making them one of the more challenging tumors to identify.
2. *Meningioma:* The meningioma images generally present well-defined, rounded masses, often appearing more uniform in texture compared to gliomas.
3. *Pituitary Tumor:* Images in this class usually feature smaller, more localized masses near the base of the brain, often showing up as distinct from the surrounding tissue.
4. *No Tumor:* These images do not contain any masses or abnormalities.

**Figure 4:**
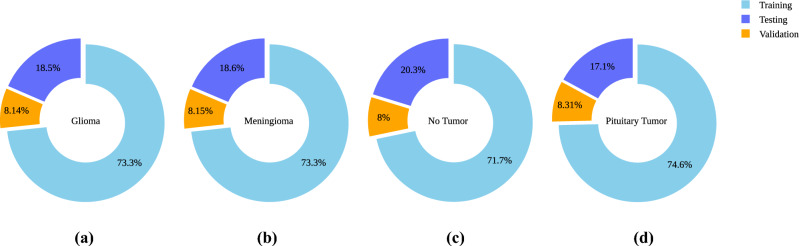
Distribution of classes in training, validation, and testing set. This figure illustrates the class-wise distribution of the “Brain Tumor MRI Dataset” across training, testing, and validation subsets. (**a**) Glioma, (**b**) Meningioma, (**c**) No Tumor, and (**d**) Pituitary Tumor, each depicting the proportion of images allocated to each subset within their respective classes.

In addition to the dataset distribution, the dataset is divided into training, testing, and validation subsets. Figure 4 displays the data distribution for each category. This dataset has been chosen due to its large number of high-quality images. It makes the preprocessing stage much more efficient. Furthermore, the dataset’s modest balance among classes prevents the model from biasing toward any majority class, ensuring fair and accurate performance across categories.

### Data pre-processing

**Figure 5:**
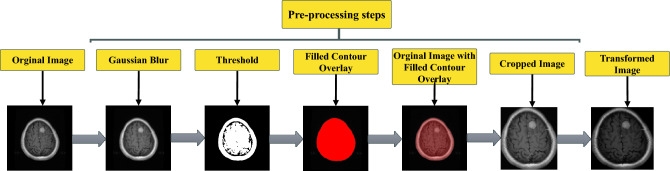
Data flow diagram of preprocessing steps.

MRI images are pre-processed to enhance feature extraction. The first step is to resize the images to 128x128 dimensions. Further pre-processing steps include converting the images to grayscale. Figure 5 provides a visual representation of the data pre-processing steps through a flow diagram. The methodology for this preprocessing can be described as a sequence of transformative actions:

#### Gaussian blur

In medical image processing, the presence of high-frequency noise can hamper the accuracy of subsequent analyses and interpretations. Here, we use Gaussian blur to smooth images and reduce detail and noise. This action is based on the Gaussian function:

9 $$\begin{aligned} GB(a, b) = \frac{1}{2\pi \sigma ^2}e^{-\frac{a^2 + b^2}{2\sigma ^2}} \end{aligned}$$

where $$GB(a, b) $$ represents the value of the Gaussian kernel at position $$ (a, b) $$. For our research, we chose a kernel size of $$ 11 \times 11 $$. The standard deviation $$ \sigma $$ is internally determined by the processing software, following the relation:

10 $$\begin{aligned} \sigma = \frac{n - 1}{6} \end{aligned}$$

Here, $n$ indicates the kernel dimension. A convolution operation is applied to the images using this kernel, yielding a blurred outcome. The purpose of this blurring is to decrease noise while preserving crucial image details, laying the groundwork for ensuing image analysis tasks.

#### Adaptive thresholding

Following the Gaussian blur, adaptive thresholding is applied. This method helps to separate brain regions from the background by highlighting specific regions of interest. Otsu’s method is used to autonomously determine the optimal threshold. This method is effective because it maximizes inter-class variation, which can be shown mathematically by:

11 $$\begin{aligned} \sigma ^2(T) = \omega _1(T) \omega _2(T) [\mu _1(T) - \mu _2(T)]^2 \end{aligned}$$

where:

- $\sigma ^2(T)$ is the inter-class variance at a given threshold $T$.
- $\omega _1(T)$ and $\omega _2(T)$ are the class probabilities for the two classes separated by the threshold.
- $\mu _1(T)$ and $\mu _2(T)$ are the class means for the two classes.

In Otsu’s method, the aim is to find the threshold $T$ that maximizes this inter-class variance. This typically results in a threshold that separates the foreground and background in an image.

#### Filled contour overlay

After applying thresholding, the next phase involves identifying contours. Contours are the boundaries separating the regions of interest (like tumors) from the rest of the image. In computer vision, contours are primarily defined as the boundaries of binary objects, differentiating the object from the background.

Our goal is to separate the brain portion from the MRI scan by removing unnecessary details and noise from MRI images. It allows the model to focus solely on the brain region. To achieve this, a minimum size threshold is set for contours. If we look at the training data, we can see that the brain represents a major portion of the MRI image. The contour area value of 1000 is used to identify larger contours. By setting this threshold, our method improves the accuracy of contour detection and lowers the chance of false positives caused by background features or minor variations that might not be medically important. This ensures that our segmentation process focuses on obtaining important parts of the brain’s structure while rejecting non-essential information.

#### Original image with filled contour overlay

To get a clearer perspective, the identified contours are put on top of the original picture. The process of overlaying can be viewed as a bitwise operation. The contours are put on top of the original picture and make it possible to visually identify important areas apart from their surroundings.

#### Cropped image

A bounding rectangle is created by using the largest contour (the region of the brain). This rectangle’s dimensions, (*x*, *y*, *w*, *h*), where *x*, *y* are the top-left coordinates and *w*, *h* are width and height, allow the image cropping to focus on this essential region.

#### Transformed image

After cropping, the pixel values of the cropped image are normalized to the range [0, 1]. This ensures a consistent intensity distribution. Thereafter, the image undergoes a power-law (or beta) transformation to emphasize its features. Mathematically, this transformation is depicted as:

12 $$\begin{aligned} P = k \times Q^{\beta } \end{aligned}$$

where:

- $P$ denotes the modified pixel value,
- $Q$ represents the original pixel value,
- $k$ is a proportionality constant, and
- $\beta$ is the transformation parameter, set to 1.5 in this study.

The power-law transformation emphasizes certain brightness levels within the standardized image. This nonlinear process brings out small details that are important for accurately finding and separating tumor areas. The image’s pixel values revert to the normal range [0, 255] after the transformation.

#### Data augmentation

A data augmentation method is employed to add diversity to the training sample. Table 1 shows the techniques that are used and the parameter ranges. These methods are chosen to focus on distinct aspects of image variability. This makes the model more applicable to real-life situations.

**Table 1:** Data augmentation techniques and their parameter ranges.

Augmentation technique	Range
Rotation	0–25 degrees
Horizontal flip	Enabled
Shear	0–0.2
Zoom	0–0.2
Width shift	– 0.2 to 0.2
Height shift	− 0.2 to 0.2

##### Rotation

Image angles were randomly changed between 0 and 25 degrees to make the model less sensitive to how things are positioned. This strategy makes it easier for the model to recognize features in a variety of orientations. An image’s rotation can be shown as a 2D affine transformation.

13 $$\begin{aligned} \begin{bmatrix} x' \\ y' \end{bmatrix} = \begin{bmatrix} \cos (\theta ) &{} -\sin (\theta ) \\ \sin (\theta ) &{} \cos (\theta ) \end{bmatrix} \begin{bmatrix} x \\ y \end{bmatrix} \end{aligned}$$

where $x, y$ are the original coordinates in the image, $x', y'$ are the coordinates after rotation, and $\theta$ is the angle of rotation.

##### Horizontal flip

A horizontal flip augmentation is employed to improve the model’s ability to understand symmetry. This makes it possible for the model to recognize features in both their original and reversed states. Horizontal flipping is a simple operation that inverts the pixel values across the vertical axis.

14 $$\begin{aligned} I_{\text {flipped}}(x, y) = I(-x, y) \end{aligned}$$

In this equation, $I_{\text {flipped}}(x, y)$ represents the pixel value at position $(x, y)$ in the flipped image, and $I(-x, y)$ denotes the pixel value at the mirrored position in the original image.

##### Shear transformation

Shear transformations between 0 and 0.2 are used to make the model more flexible in the face of small changes that happen in real-world data. Shearing can be represented as:

15 $$\begin{aligned} \begin{bmatrix} x' \\ y' \end{bmatrix} = \begin{bmatrix} 1 &{} \lambda \\ 0 &{} 1 \end{bmatrix} \begin{bmatrix} x \\ y \end{bmatrix} \end{aligned}$$

where $x, y$ are the original coordinates in the image, $x', y'$ are the coordinates after the shear transformation, and $\lambda$ is the shearing factor, ranging from $0$ to $0.2$.

##### Zoom

Zoom transformations between 0 and 0.2 have been added to the model so that it can handle changes in the size of objects. This improves the model’s ability to see features at different levels of magnification. Zooming can be expressed as:

16 $$\begin{aligned} \begin{bmatrix} x' \\ y' \end{bmatrix} = \begin{bmatrix} \alpha &{} 0 \\ 0 &{} \beta \end{bmatrix} \begin{bmatrix} x \\ y \end{bmatrix} \end{aligned}$$

where $x, y$ are the original coordinates in the image, $x', y'$ are the coordinates after applying the zoom transformation, and $\alpha , \beta$ are the scaling factors for width and height, respectively, ranging from $1$ to $1.2$ to represent a zoom range of $0$ to $0.2$.

##### Width shift and height shift

Width shift and height shift both involve the horizontal and vertical translation of the image. These changes help the model adapt to changes in where objects are in the frame. In cases where the tumor isn’t always in the middle of the brain, this is helpful for finding the tumor.

The mathematical expression for these transformations is:

17 $$\begin{aligned} \begin{bmatrix} x' \\ y' \end{bmatrix} = \begin{bmatrix} x \\ y \end{bmatrix} + \begin{bmatrix} \Delta x \\ \Delta y \end{bmatrix} \end{aligned}$$

In this equation, $x, y$ are the original coordinates in the image, $x', y'$ are the coordinates after applying the width and height shift transformations, and $\Delta x, \Delta y$ are the shift values for width and height, respectively. Shifts from $-0.2$ to $0.2$, normalized by input image dimensions, add randomization, and make it easier for the model to adapt to new data.

The combined application of these data augmentation techniques increases the dataset’s diversity and contributes to the model’s robustness and generalizability.

### Parameter setting

In the training phase of the proposed model, we adjust some parameters to achieve the best results. We focus on adjusting hyperparameters such as the number of epochs, optimizers, and learning rates. For classification among four classes, we employ the softmax activation function. All these parameter settings are displayed in Table 2.

**Table 2:** Summary of optimized hyperparameter settings.

Parameter	Optimized value
Number of epochs	50
Learning rate	0.00001
Optimizer	Adam
Loss function	Sparse categorical crossentropy
Batch size	32

### Evaluation metrics

To assess the proficiency of NeuroNet19 in identifying BTs, multiple evaluation metrics are utilized, each addressing a distinct aspect of its performance.

#### Accuracy

The accuracy is a measure of how well the model works overall. Accuracy shows how well the model’s predictions match up with the real results. It is calculated using the formula:

18 $$\begin{aligned} Accuracy = \frac{{TP + TN}}{{TP + TN + FP + FN}} \end{aligned}$$

The constituents of this equation are as follows:

- *True Positives (TP)*: Instances in which the model correctly categorizes the presence of a tumor.
- *True Negatives (TN)*: Instances in which the model correctly indicates the absence of a tumor.
- *False Positives (FP)*: Cases in which the model erroneously flags a healthy scan as a tumor.
- *False Negatives (FN)*: Cases in which the model incorrectly overlooks a tumor, classifying it as a healthy scan.

#### Precision

This evaluates how well the model can reduce false positives, which shows how reliable it is at confirming tumor cases. It is defined as:

19 $$\begin{aligned} \text {Precision} = \frac{\text {TP}}{\text {TP} + \text {FP}} \end{aligned}$$

#### Recall

Assesses the model’s ability to identify all actual tumor cases and is defined as:

20 $$\begin{aligned} \text {Recall} = \frac{\text {TP}}{\text {TP} + \text {FN}} \end{aligned}$$

#### F1-score (F1)

The F1-Score balances the trade-offs between precision and recall. It is the harmonic mean of these two metrics and is calculated as:

21 $$\begin{aligned} \text {F1-Score} = \frac{2 \times (\text {Precision} \times \text {Recall})}{\text {Precision} + \text {Recall}} \end{aligned}$$

The F1-Score is especially useful when dealing with imbalanced classes, where both false positives and false negatives are consequential.

#### Cohen’s Kappa coefficient ($\kappa$)

Cohen’s Kappa calculates how much the model’s predictions and the real labels agree, taking into account the possibility of random agreement. Accuracy can be too high when the classes in a dataset aren’t spread out evenly. ($\kappa$) gives a more accurate picture by considering the chance of random agreement. A higher $\kappa$ value means that the model agrees strongly with the real labels. This makes $\kappa$ an effective way to measure how reliable classification models are. This is particularly important in medical diagnostics, where both types of errors–false positives and false negatives–carry significant consequences. It is mathematically formulated as:

22 $$\begin{aligned} \kappa = \frac{(p_o - p_e)}{(1 - p_e)} \end{aligned}$$

This equation shows that the observed agreement $p_o$ is the actual proportion of the agreement between the raters, and the expected agreement $p_e$ is the likelihood that the raters will agree by chance. This number, $\kappa$, tells how much the real agreement is stronger than chance: 1 means there is a perfect agreement, 0 means there is a random agreement, and negative numbers show systematic disagreement.

### Experimental setup

The experiment is conducted on Google Colab by utilizing its Tesla T4 GPU for enhanced computational efficiency. We employ a 3-fold cross-validation strategy for robust model evaluation. For the best model, checkpoints are set up to save model parameters when validation accuracy improves. In addition, we record each fold’s training and validation data to observe how the model improves over time.

## Experimental exploration

### Evaluative overview of NeuroNet19 across diverse configurations

We conducted an ablation study to get the best results from our model. Here, we train our proposed model, NeuroNet19 by adjusting one parameter at a time. Specifically, we experiment with varying the optimizer, increasing the total number of epochs, and adjusting the learning rate. This study provides valuable insights into how each parameter contributes to the overall performance of the model. The findings of this ablation study are presented in Table 3. The table presents an in-depth analysis by demonstrating the performance metrics of models that were trained with a variety of hyperparameters.

The model with the Adam optimizer, a learning rate of 0.00001, and training over 50 epochs shows superior performance, achieving the best accuracy, precision, recall, F1 score, and Cohen’s Kappa coefficient. This optimal balance between learning efficiency and model generalization ensures accurate and robust predictive results. On the other hand, models using the Adadelta optimizer performed significantly worse across all evaluated metrics. In particular, models implemented with a learning rate of 0.00001 are not optimal. This shows Adadelta’s inherent limitations in helping find the best solution in the given experimental situations.

**Table 3:** Ablation study of NeuroNet19 model.

Optimizer	Learning rate	Epoch	Accuracy (%)	Precision (%)	Recall (%)	F1 (%)	Cohen’s Kappa	Loss
Adam	0.00001	20	98.6	98.6	98.5	98.5	98.1%	0.052
	0.0001	20	98.5	98.5	98.4	98.5	98%	0.064
	0.001	20	30	7	25	11	0	1.379
	0.0001	30	98.3	98.2	98.2	98.2	97.8%	0.055
	0.00001	30	97.4	97.3	97.2	97.2	96.5%	0.193
	0.001	30	30.8	7	25	11	0	1.380
	0.0001	40	97.4	97.5	97.3	97.4	96.6%	0.052
	0.00001	40	99	99	99	99	98.7%	0.049
	**0.00001**	**50**	**99.3**	**99.2**	**99.2**	**99.2**	**99%**	**0.040**
	0.0001	50	98.7	98.6	98.5	98.6	98.2%	0.052
RMSprop	0.00001	20	96.6	96.6	96.3	96.3	96.6%	0.184
	0.0001	20	95.1	95.1	94.7	97.8	94.3%	0.221
	0.001	20	30.8	7	25	11	0	1.380
	0.00001	30	97.8	97.9	97.6	97.7	97.1%	0.143
	0.0001	30	96.7	96.6	96.6	96.6	95.5%	0.297
	0.001	30	27.2	25.3	28.5	15.7	0	1.381
	0.00001	40	98.5	98.4	98.4	98.4	98.5%	0.198
	0.00001	50	98.7	98.7	98.7	98.7	98.3%	0.097
	0.0001	40	98.4	98.4	98.3	98.3	97.9%	0.088
	0.0001	50	96.6	96.6	96.4	96.5	95.4%	0.185
Adagrad	0.00001	20	85.3	85.2	84.5	84.2	80.3%	0.455
	0.0001	20	94.8	94.8	94.4	94.4	94.8%	0.179
	0.001	20	97.7	97.6	97.6	97.6	97%	0.085
	0.00001	30	86.4	85.8	85.7	85.4	81.7%	0.432
	0.0001	30	95.6	95.5	95.3	95.3	94.1%	0.168
	0.001	30	97.2	97.2	97	97.2	96.3%	0.145
	0.00001	40	86.8	86.6	86	85.8	82.2%	0.435
	0.00001	50	87.4	87.3	86.7	86.5	83.2%	0.378
	0.0001	40	95.1	95.2	94.8	94.8	93.3%	0.222
	0.0001	50	96.1	95.9	95.8	95.8	94.7%	0.180
SGD	0.00001	20	85.5	84.7	84.6	84.4	80.5%	0.453
	0.0001	20	90.2	90.8	89.5	89.5	86.2%	0.346
	0.001	20	97	96.9	96.8	96.8	96%	0.140
	0.00001	30	85.5	85	84.8	84.3	80.6%	0.441
	0.0001	30	95.1	95	94.7	94.8	93.4%	0.169
	0.001	30	98.4	98.3	98.2	98.3	97.8%	0.068
	0.00001	40	88.4	88	87.9	87.8	84.5%	0.362
	0.00001	50	87.9	88.2	87.1	87.1	83.8%	0.423
	0.0001	40	94.6	94.6	94.3	94.2	92.8%	0.201
	0.0001	50	95.6	95.4	95.3	95.3	94.1%	0.205
Adadelta	0.00001	20	51.5	49.9	50.5	48.5	34.9%	1.257
	0.0001	20	81.9	81.4	80.9	80.5	75.7%	0.574
	0.001	20	94.5	94.4	94.2	94.2	92.7%	0.170
	0.00001	30	57.7	56.1	55.7	55.7	43.2%	1.039
	0.0001	30	82.6	82.6	81.7	81.1	76.7%	0.600
	0.001	30	89.4	90.1	88.5	88.5	85.7%	0.469
	0.00001	40	64.8	62.9	62.4	62	52.4%	0.970
	0.00001	50	65.1	65.2	63.3	62.8	53%	0.936
	0.0001	40	86.9	86.3	86.3	86	82.4%	0.396
	0.0001	50	84.1	84.1	83.3	82.8	78.6%	0.521

Significant values are in bold.

**Figure 6:**
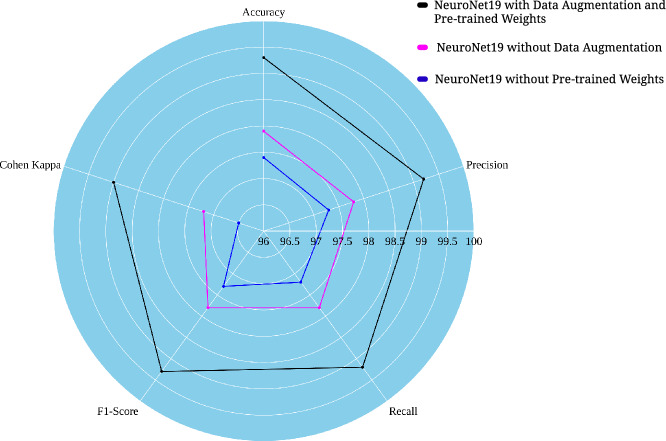
Performance metrics for various models.

The performance of NeuroNet19 is evaluated in three different configurations. These configurations are as follows: with pre-trained weights and data augmentation; without pre-trained weights; and without data augmentation. Precision, recall, F297-Score, and Cohen’s Kappa are used to evaluate each version’s strengths and weaknesses. Figure 6 is a radar chart that displays how well NeuroNet19 works in all three configurations. When combined with data augmentation and pre-trained weights, the NeuroNet19 model performs well in all metrics. It shows high accuracy (99.3%), precision (99.2%), recall (99.2%), F1-Score (99.3%), and CKC score (99.0%). According to this result, the model probably performs best when it is trained with a diverse dataset through data augmentation and initialized with pre-trained weights. Training the model without data augmentation lowers accuracy (97.9%), precision and recall (97.8%), F1-Score (97.8%), and CKC (97.2%). This significant decrease emphasizes the importance of data augmentation in improving data quality and quantity. NeuroNet19 without pre-trained weights has the lowest accuracy (97.4%), precision (97.2%), recall (97.3%), F1-Score (97.3%), and CKC (96.5%). This suggests that pre-trained weights enhance model prediction and classification.

**Figure 7:**
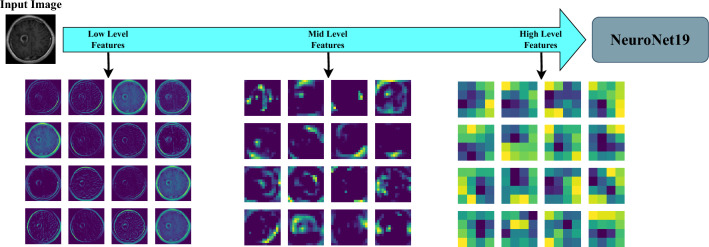
Performance metrics for various models.

NeuroNet19’s feature maps illustrate a hierarchical learning representation of the proposed model in Figure 7. The feature maps can be divided into three different levels: low-level, mid-level, and high-level. It shows how well the model can understand a diverse set of features, ranging from the most complicated to the most fundamental. The low-level filters capture the low-level features, which include features like edges and textures. It can be used as a building block to make more complicated features. As it progresses to the mid-level features, it begins to recognize more complicated patterns, such as shapes and object components. The low-level representations and high-level features are used to identify complex data things like object classes and complex inter-object relationships.

**Figure 8:**
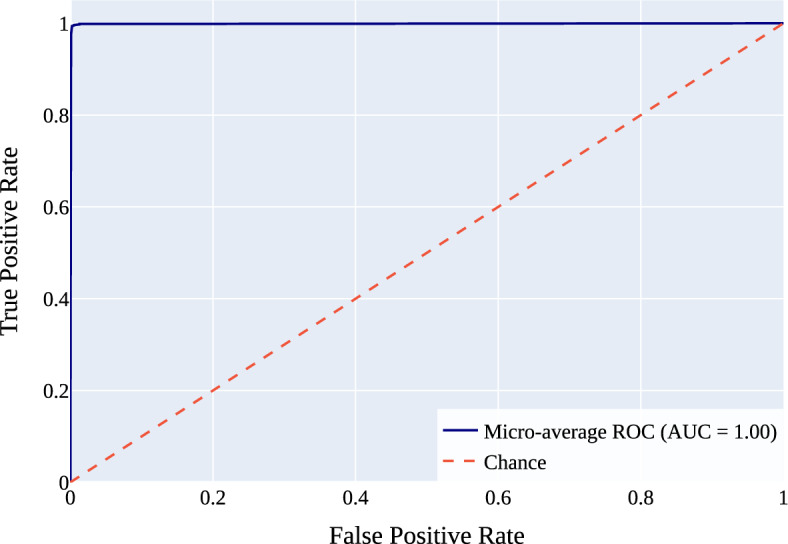
Micro-average ROC curve for NeuroNet19.

Figure 8 represents the Micro-Average Receiver Operating Characteristic (ROC) curve for NeuroNet19. Micro-average combines all class outcomes instead of computing metrics separately. This method treats our classification problem as a binary task. It considers every true positive, false positive, true negative, and false negative for all classes, including “Glioma,” “Meningioma,” “No Tumor,” and “Pituitary Tumor.” After combining these outcomes, the ROC curve is generated to evaluate NeuroNet19. The curve in Fig. 8 illustrates the true positive rate (TPR) and false positive rate (FPR) at various threshold values. The ratio of true positives to total positives (true positives plus false negatives) is the true positive rate (TPR), or sensitivity. A false positive rate (FPR) is the number of false positives divided by the total number of actual negatives. The micro-average ROC curve shows the model’s ability to discriminate between positive and negative cases across all classes. We may learn about our model’s efficacy by looking at the AUC value (AUC value = 1), the shape of the curve, and how close it is to the top-left corner. A curve that fits closely to the upper left corner and an AUC number that is 1 show that NeuroNet19 is very good at identifying the difference between classes. This means that our model is capable of correctly identifying various forms of tumors and separating them from non-tumor cases.

### Comparative analysis of NeuroNet19 and established models

In this study, a comparative analysis is conducted where we compare our proposed model, NeuroNet19 with several others such as ResNet50, VGG16, VGG19, MobileNet, MobileNetV2, ViT-B-32, and DenseNet121. The performance of each model is evaluated by utilizing a wide variety of evaluation metrics and illustrative visualizations.

**Figure 9:**
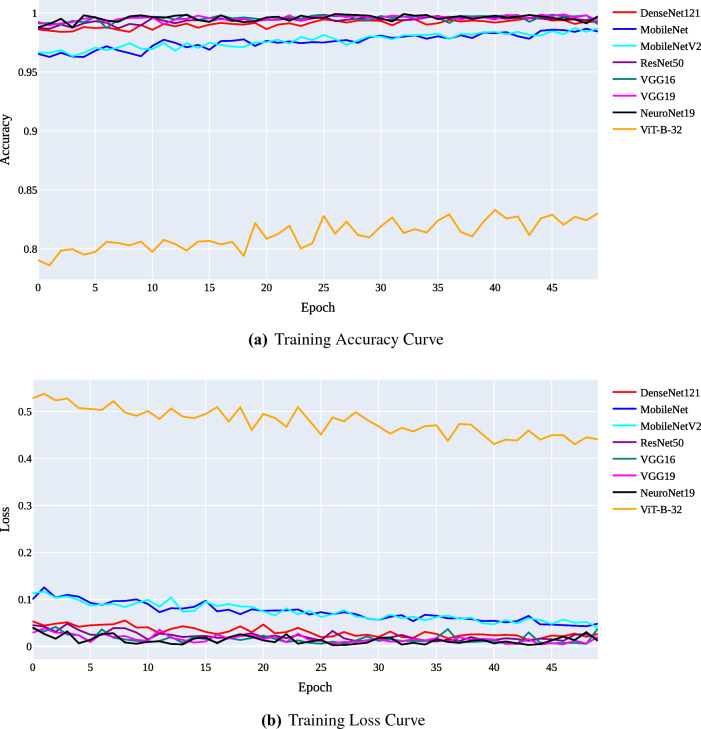
Training curves for all applied models.

**Figure 10:**
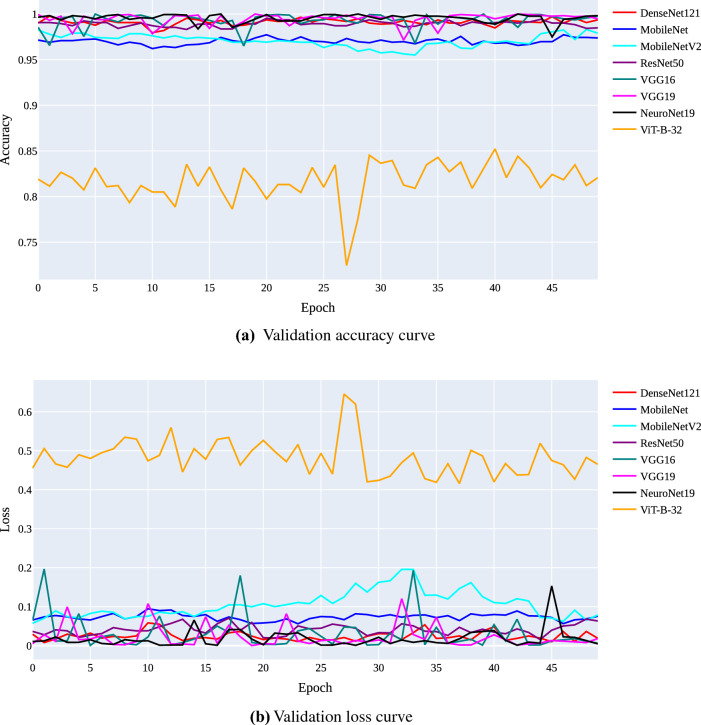
Validation curves for all applied models.

The training and validation curves for each model are shown in Figs. 9 and 10, respectively. Figure 9a and b illustrate the training accuracy and loss curves, while Fig. 10a and b illustrate the validation accuracy and loss curves. The X-axis on the accuracy curves shows the number of epochs, and the Y-axis shows the accuracy (the proportion of accurate predictions made on the validation set). From Fig. 9a, we can see that during the training process, the accuracy of all models continues to increase. The accuracy of NeuroNet19, DenseNet121, ResNet50, VGG16, and VGG19 is rapidly and simultaneously improving. In contrast, MobileNet continues to lag despite its improvements, while ViT-B-32 is currently in last place. According to validation accuracy, NeuroNet19 performs the best, followed by VGG16, VGG19, ResNet50, and DenseNet121. The performance of MobileNetV2 is better than MobileNet, while ViT-B-32 continues to underperform.

**Figure 11:**
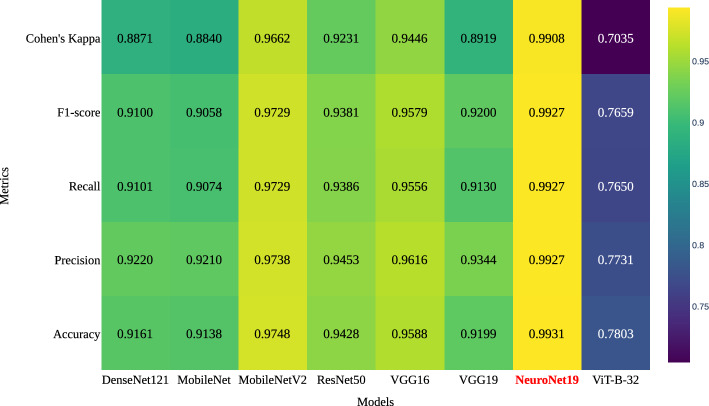
Heatmap illustrating performance metrics on the test dataset.

A heatmap is presented in Fig. 11, which illustrates the five main performance metrics of accuracy, precision, recall, F1 score, and Cohen’s Kappa score. These metrics are calculated using the test dataset. This provides a comprehensive evaluation of each model’s overall performance. In this heatmap, each model is assessed based on the mentioned metrics. The models include DenseNet121, MobileNet, MobileNetV2, ResNet50, VGG16, VGG19, NeuroNet19, and ViT-B-32. The color gradient in the heatmap ranges from 0.70 to 1. Here, 1 represents the highest value in the metric.

NeuroNet19 shows exceptional performance on all measures; it achieves an accuracy of 99.3%, precision of 99.2%, recall of 99.2%, F1-score of 99.2%, and Cohen’s Kappa of 99%, demonstrating balanced and outstanding performance in classification and reliability. In contrast, the performance of the ViT-B-32 model is much poorer than other models, with an accuracy of 78%, a precision of 77.3%, a recall of 76.5%, an F1-score of 76.5%, and a Cohen’s Kappa score of 70.3%, respectively. DenseNet121, MobileNet, MobileNetV2, ResNet50, VGG16, and VGG19 exhibit intermediate performance levels, with MobileNetV2 showing notable improvement over MobileNet, particularly in precision, recall, and F1-score.

**Figure 12:**
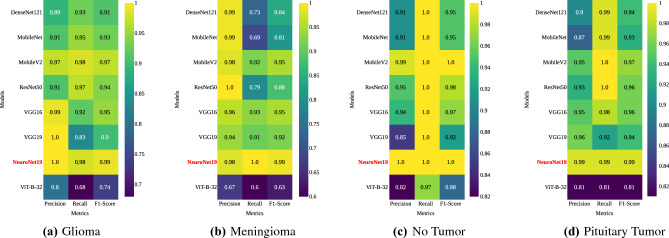
Class-wise heatmaps: (**a**) Glioma; (**b**) Meningioma; (**c**) No Tumor; (**d**) Pituitary Tumor, depicting precision, recall, and F1-score for each model.

Figure 12 expands the analysis by presenting class-wise heatmaps. These heatmaps focus on different tumor classes, such as glioma, meningioma, no tumor, and pituitary Tumor. It also compares the performance of different models in terms of precision, recall, and F1-score for each specific class. Observing the heatmap for Glioma depicted in Fig. 12a, both NeuroNet19 and VGG19 stand out as having a precision of 1.0. Notably, NeuroNet19 secures the leading position with an F1-Score of 0.99, emphasizing its proficiency in the identification of gliomas. On the other hand, ViT-B-32 appears less adept for this specific glioma class. In the Meningioma heatmap Fig. 12b, NeuroNet19 maintains its superior recall and F1-Score of 0.99. This shows how reliable it is at finding meningiomas, while ViT-B-32 once again falls behind with lower numbers. In the context of no tumor conditions, as displayed in Fig. 12c, both NeuroNet19 and MobileNetV2 showcase exemplary performance across all evaluation metrics, proving their capability of distinguishing tumor-absent scenarios. Here, ViT-B-32 manages a respectable recall of 0.97. Finally, when focusing on the pituitary tumors, as seen in Fig. 12d, NeuroNet19 maintains its high level of performance, whereas ViT-B-32 continues to deliver a performance that is consistently below average. These class-specific heatmaps provide a clear portrayal of each model’s diagnostic ability across a variety of BTs. Under this perspective, NeuroNet19 continuously performs well, but ViT-B-32 highlights some areas of difficulty.

**Figure 13:**
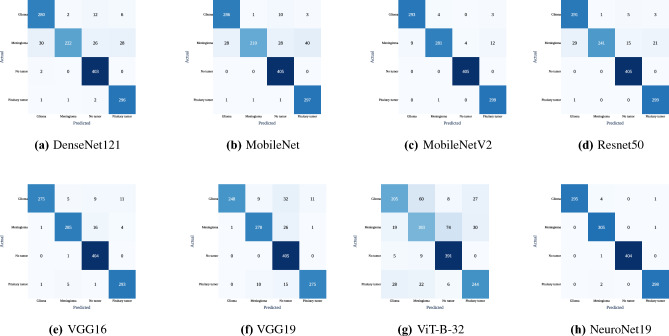
Confusion matrices for various models.

In addition to the above metrics, confusion matrices are compiled for each model. These matrices provide a visual summary of true and false classifications as well as insights into the performance of each model across a variety of classes, such as glioma, meningioma, no tumor, and pituitary tumor. The confusion matrices in Fig. 13 correspond to ResNet50, VGG16, VGG19, MobileNet, MobilenetV2, DenseNet121, ViT-B-32, and NeuroNet19, respectively. NeuroNet19 classified 295 cases of glioma, 305 cases of meningioma, 404 cases of no tumor, and 298 cases of pituitary tumor with negligible errors, demonstrating its precision and reliability. Other models, including MobileNetV2 and DenseNet121, etc., shows misclassifications, particularly between Glioma and Meningioma. ViT-B-32, on the other hand, displays substantial inaccuracies and misclassifications across all tumor types, with only 205 correct classifications for glioma and 183 for meningioma, emphasizing its limitations. NeuroNet19 has the potential to be the best model in medical diagnostics because it consistently outperforms other models in precision, recall, and overall accuracy.

### Comparative analysis of model features and interpretability across models

t-Distributed Stochastic Neighbor Embedding (t-SNE) is an unsupervised, non-linear dimensionality reduction technique that allows the visualization of high-dimensional data in a low-dimension space. By transforming the n-dimension into two or three dimensions, the t-SNE maintains the relative similarities between data points. The primary goal is to see if the features in this 2D space cluster closely concern each tumor class (e.g., glioma, meningioma, no tumor, pituitary Tumor). Close intra-class clustering indicates that the model can effectively distinguish these classes from one another. Figures “reffig:t-SNE” use t-SNE (t-Distributed Stochastic Neighbor Embedding) visualizations to examine the feature maps produced by various models for classifying various types of tumors.

**Figure 14:**
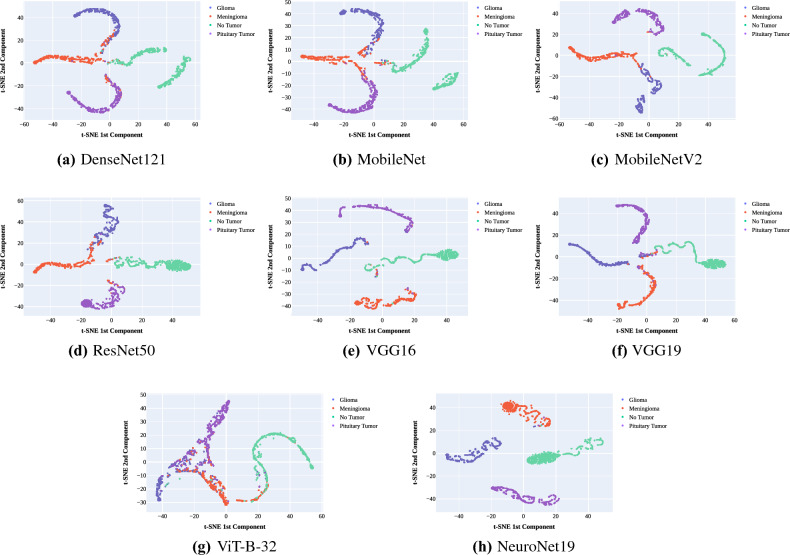
Feature Clustering analysis: t-SNE Visualization for tumor classification.

In Fig. 14a–c we observe that the feature data points corresponding to the “No Tumor” class are scattered in the middle of the plot. This indicates that the model struggles to capture distinct features specific to healthy brain tissue. Figure 14a–f all show overlap between different class feature clusters. Specifically in Fig. 14g, glioma, meningioma, and pituitary tumor feature clusters meld into one another, while only the no-tumor features manage to form a distinct cluster. Figure 14h, on the other hand, stands out by showing very tight and well-separated clusters. This shows how good NeuroNet19 is at finding and recognizing very different features in different tumor types. While there are still a few instances where points from one class are found within the cluster of another class, this occurrence is minimal compared to the other models evaluated.

### Features explainability analysis

**Figure 15:**
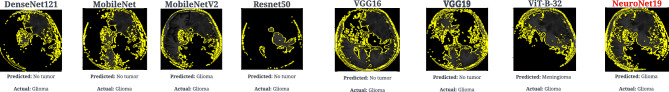
LIME-based feature importance analysis.

In evaluating multiple BT classification models, we use LIME (local interpretable model-agnostic explanations) to learn about image features or regions that predict the tumors well. Lime takes the model as input and generates explanations about feature contributions when predicting an individual example. This method displays significant features of the images by highlighting regions that have a high degree of alignment between the model’s focus and features that are clinically relevant. This suggests the method could be reliable in the clinic. Figure 15 shows the important areas that affect predictions across models and help us understand how decisions are made.

In this context, we compare the predictions of different models on a glioma image. This image has been chosen because it is misclassified by all models except Neuronet19 and MobileNetV2. Because we know that LIME’s interpretations can change from one execution to the next, we ran the algorithm 20 times to make sure that the interpretations are always the same. To get a more consistent and reliable interpretation, we then find the mean of the results from all of these different runs. We use a superpixel level of 3000 to make sure that we can carefully look at the features that affected the models’ predictions. This allows us to get a full picture of the features that mattered.

## Discussion

The NeuroNet19 model demonstrates remarkable performance in the classification of BTs. It outperformed many existing models in the field with an exceptional accuracy rate of 99.33%. Such accuracy is vital in medical applications where precision and reliability are essential. In addition, the model has impressive precision and recall scores of 99.2%, which demonstrates that it can accurately make identifications. NeuroNet19 also lowers the number of false positives and false negatives. The F1-Score and CKC scores of 99.3% and 99.9%, respectively, reinforce the model’s ability to handle imbalanced datasets.

When the NeuroNet19 model is compared to existing approaches in BT classification, it is clear that this method has several advantages. In the past, traditional image processing methods and transfer learning approaches were frequently used. Despite making important contributions, they often face problems with scale variance, data that isn’t balanced, and the stability of their predictions. A unique feature of the NeuroNet19 approach is the integration of iPPM into its architecture. This feature allows the model to capture both macro- and micro-features in brain MRI images. As a result, iPPM’s multi-scale feature capture improves the ability to distinguish intricate tumor patterns and variations in tumor classes. This solves a significant problem in the classification of BTs, which is the significant variation in tumor sizes and locations that can be seen in MRI scans.

**Figure 16:**
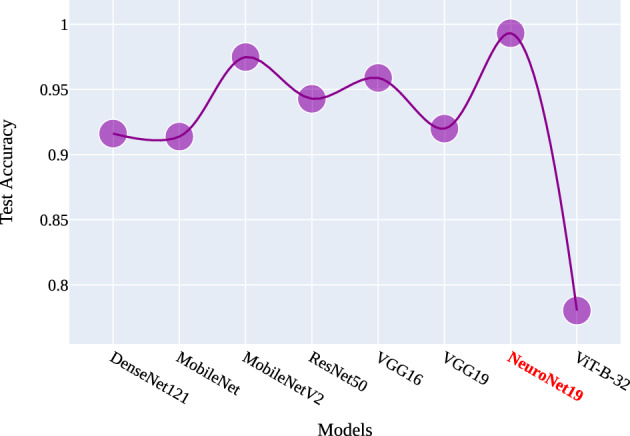
Comparison of models test accuracy.

**Figure 17:**
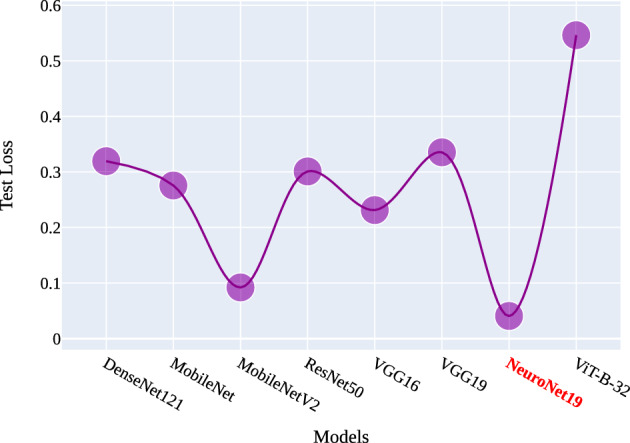
Comparison of models test loss.

Figures 16 and 17 illustrate the comparative accuracy and loss across various models. The accuracy curve in Fig. 16 reveals that the model with the highest accuracy is NeuroNet19, followed by MobileNetV2, VGG16, ResNet50, VGG19, DenseNet121, MobileNet, and VIT-B-32, in that order. On the other hand, the loss curve in Fig. 17 shows that NeuroNet19 has the lowest test loss, followed by MobileNetV2, VGG16, MobileNet, ResNet50, DenseNet121, and VIT-B-32. Notably, the proposed NeuroNet19 model stands out in both accuracy and loss metrics. The comparison between previous research studies is presented in Table 4.

**Table 4:** Summary of models, datasets, accuracy, precision, recall, and F1-score achieved in the BT detection literature.

Study	Year	Model used	Dataset	Accuracy (%)	Precision	Recall	F1
^14^	2022	(SVM) using FLAIR for HGG	BRATS 2018	96.19	95.8%	85.1%	87%
^13^	2022	Nine-layered CNN model with hybrid division histogram equalization	BRATS datasets (2013–2018)	99.06	–	–	–
^19^	2023	Weighted average ensemble deep learning model	The Cancer Genome Atlas (TCGA)	98	98.25%	–	98.5%
^20^	2022	LeaSE+DARTS architecture	Brain tumor classification (MRI)^30^	90.61	91.49%	91.5%	91.48%
^21^	2022	EfficientNet-B0	Brain tumor^31^	98.87	99.4%	99.5%	98.9%
^22^	2023	CNN with GNN	Brain tumor classification (MRI)^30^	95.01	–	–	–
^23^	2024	Sine cosine Archimedes optimization algorithm (SCAOA) based DenseNet	BRATS 2020 and figshare	93.0	–	–	–
^24^	2024	ARM-Net	MBTD and BraTS 2020	96.64	–	–	–
^25^	2023	CNN model with explainable artificial intelligence (XAI)	Custom dataset	96	–	–	–
^26^	2023	ResNet50	Brain tumor dataset^32^	95.32	95.11%	95.15%	95.15%
^15^	2023	Modified ResNet50 with HOG	Kaggle	88	80%	–	–
^28^	2023	TECNN	BraTS 2018 and figshare	99.10	–	–	–
Proposed Model	-	NeuroNet19	Brain Tumor MRI dataset^29^	99.3	99.2%	99.2%	99.3%

## Conclusion

The NeuroNet19 model has established a new standard in the identification of BTs due to its remarkable accuracy. We propose NeuroNet19, which combines VGG19 and iPPM modules for multi-scale feature extraction. We also utilize LIME to learn which features are most important when it comes to predicting. Our proposed model, NeuroNet19, not only highlights the model’s advanced proficiency in classification tasks but also highlights the model’s transparency with LIME. The model analyzes the results of magnetic resonance imaging (MRI) scans to improve classification and reduce the number of false negatives, thereby ensuring that patients receive treatment that is both timely and accurate. NeuroNet19 shows outstanding performance when it comes to the analysis of MRI scans by achieving an accuracy of 99.3%, a precision and recall of 99.2%, an F1 score of 99.3%, and a CKC score of 99%. The high level of accuracy exhibited by the proposed model, NeuroNet19 results in a low rate of tumor detection errors. Therefore, patients can have confidence in receiving prompt and high-quality healthcare. NeuroNet19’s limitations include that it is trained on four different types of tumors. Therefore, it won’t be used for binary classes. New research and classifications of skin conditions are constantly being discovered in the medical field. To work on new tumor categories, Neuronet19 must be trained on that type of tumor. However, there is always room for improvement in the vast field of scientific research. Future research will concentrate on expanding the dataset and enhancing NeuroNet19’s versatility and adaptability across diverse clinical scenarios. Also, since CT scan images are becoming increasingly useful in diagnostics, efforts will be focused on improving the NeuroNet19 model for its implementation in CT scans and other imaging modalities. A web-based platform that incorporates the NeuroNet19 model will be developed as part of an effort to put this research into practice. The purpose of this strategic move is to make this cutting-edge diagnostic tool more accessible to medical professionals all over the world. This effort will open the door to a new era of diagnosing BTs. We intend to integrate federated learning into our future work to publish our model globally and facilitate its ongoing refinement through the development of an expert system.

## Data Availability

The used dataset of this study “Brain Tumor MRI Dataset” is publicly available at https://www.kaggle.com/datasets/masoudnickparvar/brain-tumor-mri-dataset/data with the DOI [10.34740/kaggle/dsv/2645886].
